# Molecular Organization and Functional Analysis of a Novel Plasmid-Borne *cps* Gene Cluster from *Lactiplantibacillus plantarum* YC41

**DOI:** 10.1128/spectrum.04150-22

**Published:** 2023-03-06

**Authors:** Jieran An, Yuchen Zhang, Zhaoer Zhao, Ran Huan, Huaxi Yi, Hui Wang, Chunguang Luan, Shengbao Feng, Heqiang Huang, Shanwen Li, Deliang Wang, Zhengyuan Zhai, Yanling Hao

**Affiliations:** a Key Laboratory of Functional Dairy, Co-constructed by the Ministry of Education and Beijing Municipality, College of Food Science and Nutritional Engineering, China Agricultural University, Beijing, China; b Key Laboratory of Precision Nutrition and Food Quality, Department of Nutrition and Health, China Agricultural University, Beijing, China; c College of Food Science and Engineering, Ocean University of China, Qingdao, China; d China National Research Institute of Food and Fermentation Industries, Beijing, China; e Qinghai Huzhu Barley Wine Co. Ltd., Haining, China; 南昌大学

**Keywords:** *Lactiplantibacillus plantarum*, capsular polysaccharide, *cps* gene cluster, plasmid, environmental stresses

## Abstract

Capsular polysaccharide (CPS) can tightly attach to bacterial surfaces and plays a critical role in protecting microorganisms from environmental stresses. However, the molecular and functional properties of some plasmid-borne *cps* gene clusters are poorly understood. In this study, comparative genomics of the draft genomes of 21 Lactiplantibacillus plantarum strains revealed that the specific gene cluster for CPS biosynthesis was observed only in the 8 strains with a ropy phenotype. Furthermore, the complete genomes showed that the specific gene cluster *cpsYC41* was located on the novel plasmid pYC41 in L. plantarum YC41. *In silico* analysis confirmed that the *cpsYC41* gene cluster contained the dTDP-rhamnose precursor biosynthesis operon, the repeating-unit biosynthesis operon, and the *wzx* gene. The insertional inactivation of the *rmlA* and *cpsC* genes abolished the ropy phenotype and reduced the CPS yields by 93.79% and 96.62%, respectively, in *L. plantarum* YC41 mutants. These results revealed that the *cpsYC41* gene cluster was responsible for CPS biosynthesis. Moreover, the survival rates of the YC41-rmlA^−^ and YC41-*cpsC*^−^ mutants under acid, NaCl, and H_2_O_2_ stresses were decreased by 56.47 to 93.67% compared to that of the control strain. Furthermore, the specific *cps* gene cluster was also confirmed to play a vital role in CPS biosynthesis in *L. plantarum* MC2, PG1, and YD2. These findings enhance our understanding of the genetic organization and gene functions of plasmid-borne *cps* gene clusters in *L. plantarum*.

**IMPORTANCE** Capsular polysaccharide is well known to protect bacteria against various environmental stresses. The gene cluster for CPS biosynthesis is typically organized in the chromosome in bacteria. It is worth noting that complete genome sequencing showed that a novel plasmid pYC41-borne *cpsYC41* gene cluster was identified in *L. plantarum* YC41. The *cpsYC41* gene cluster included the dTDP-rhamnose precursor biosynthesis operon, the repeating-unit biosynthesis operon, and the *wzx* gene, which was verified by the significantly decreased CPS yield and the absent ropy phenotype in the corresponding mutants. The *cpsYC41* gene cluster plays an important role in bacterial survival under environmental stress, and the mutants had decreased fitness under stress conditions. The vital role of this specific *cps* gene cluster in CPS biosynthesis was also confirmed in other CPS-producing *L. plantarum* strains. These results advanced a better understanding of the molecular mechanisms of plasmid-borne *cps* gene clusters and the protective functionality of CPS.

## INTRODUCTION

Lactiplantibacillus plantarum is a facultative heterofermentative lactic acid bacterium (LAB). The larger genome (~3.3 Mb) and the higher proportion of regulatory genes than those of other *Lactobacillus* species allow this species to survive in a variety of different environmental niches (1). L. plantarum has been widely used in fermented food and feed production, such as dairy, meats, vegetables, and silage (2–4). In addition, it can exhibit various health-promoting effects, including reducing cholesterol levels (5), regulating the immune system (6), alleviating memory impairment (7), and enhancing epithelial barrier function (8). However, during manufacturing or consumption, *L. plantarum* suffers from unavoidable stress such as dehydration, extreme temperatures, osmotic stress, acid, and bile salt (9). Hence, *L. plantarum* has developed various adaptive mechanisms to cope with different stresses.

Bacterial polysaccharides represent a large group of carbohydrate polymers, which are either tightly attached to the surface of the microbial cell in the form of capsular polysaccharide (CPS) or loosely attached and secreted into the environment as exopolysaccharide (EPS) (10, 11). They are well known to protect bacteria against environmental stresses by forming a physical barrier on the cell surface (12, 13). When exposed to acidic conditions, an EPS-producing strain showed 20-fold increases in viability compared with a non-EPS-producing strain in Lactobacillus paracasei NFBC 338 (14). Compared with the wild type, the survival rate of an *epsE* mutant was decreased by 10% under heat shock in Lactobacillus johnsonii FI9785 (15). It has also been reported that CPS resulted in a 20% improvement in bacterial survival in the presence of 1% bile salt in *L. plantarum* LTC-113 (16). However, in *L. plantarum* Lp90 and SF2A35B, there were no significant differences in survival rates between the wild-type strains and the corresponding *cps* deletion mutants under gastrointestinal (GI) tract-mimicking conditions (17). These results indicated that the relationship between CPS and survival strategies remains unclear, even within one species.

The CPS biosynthesis genes are organized as *cps* gene clusters in *L. plantarum* (18). The *cps* gene clusters generally include regulatory genes encoding the regulatory proteins CpsABCD, repeating-unit synthesis genes encoding glycosyltransferases, assembly machinery genes encoding Wzx and Wzy, sugar precursor synthesis genes, and genes for sugar residue modification (19). Up to now, 192 *L. plantarum* strains with complete genome sequences and 539 *L. plantarum* strains with draft genome sequences have been deposited in the NCBI database. *cps* gene clusters located in chromosomes are found in 43 *L. plantarum* strains, and the genetic organizations of the gene clusters were further characterized in 12 strains, *L. plantarum* WCFS1, ZJ316, ST-III, NC8, JDM1, P8, 16, SF2A35B, Lp90, LTC-113, SN35N, and K25 (16, 20, 21). Furthermore, plasmid-borne *cps* gene clusters have been found in 10 *L. plantarum* strains, *L. plantarum* ZJ316, 16, C410L1, HFC8, LZ227, TMW1.1623, ZJ102, LTC-113, SN35N, and K25 (16, 20, 21). Only the plasmid-borne *cps* gene cluster in *L. plantarum* LTC-113 has been well characterized (16). Thus, it will be necessary to further characterize *cps* gene clusters on plasmids in *L. plantarum*.

In this study, *L. plantarum* YC41 was isolated from sauerkraut, a traditional brine-salted Chinese fermented food. Genetic and physical experiments revealed that the *cpsYC41* gene cluster was responsible for CPS biosynthesis. Furthermore, *cpsYC41*-derived CPS also showed increased tolerance to environmental stresses, including acid, NaCl, and H_2_O_2_ stresses. These results provide insight into the molecular genetic mechanism of *cps* clusters on plasmids in *L. plantarum* and the beneficial role of CPS in bacterial survival under stress.

## RESULTS

### Eight *L. plantarum* strains formed ropy colonies.

**FIG 1:**
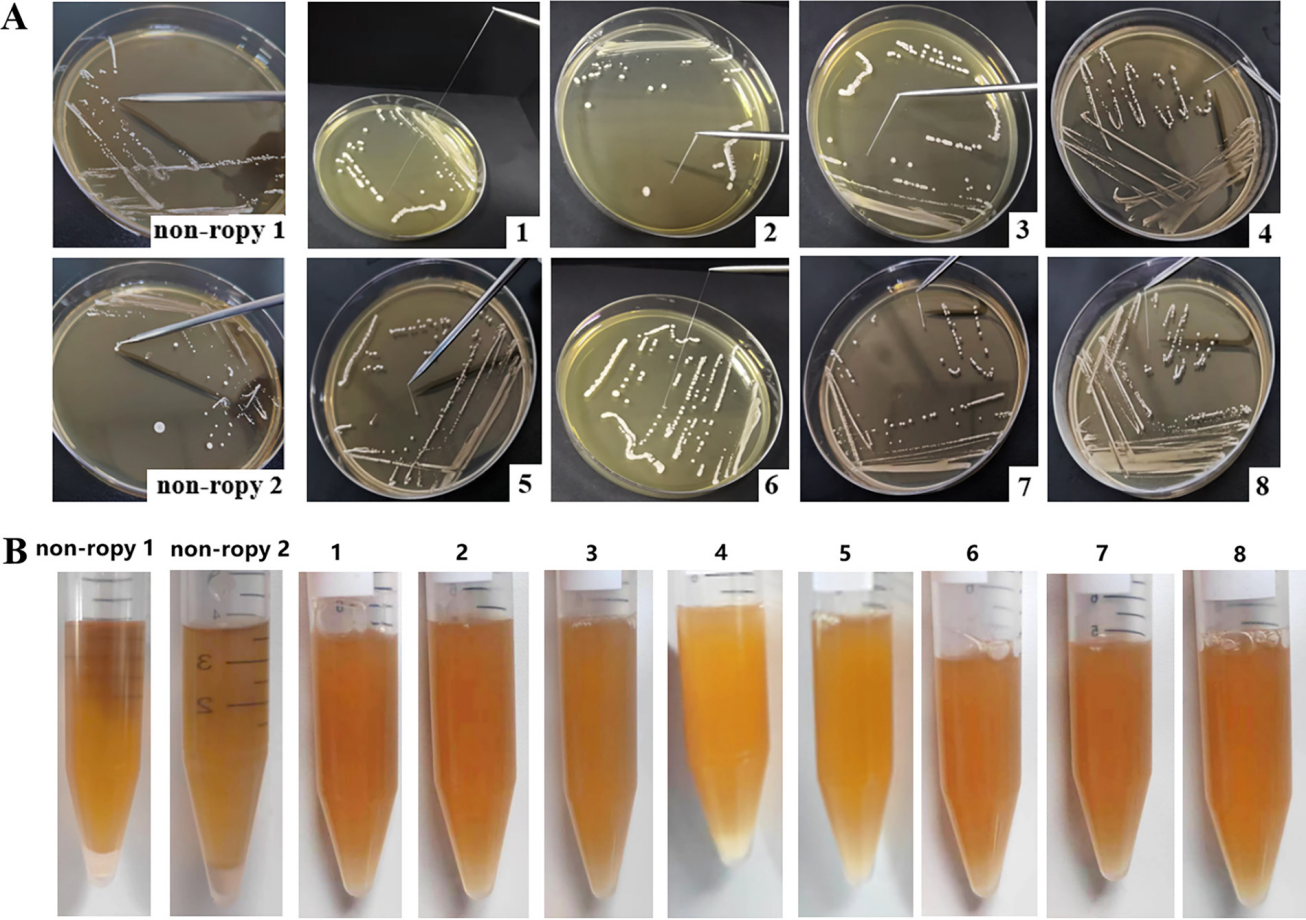
Characteristic features of *L. plantarum* strains with a ropy phenotype. (A) Detection of ropy *L. plantarum* strains on MRS agar containing 2% (wt/vol) sucrose. (B) Autoaggregation of *L. plantarum* incubated at 37°C for 20 h in MRS broth. Nonropy 1, *L. plantarum* JY; nonropy 2, *L. plantarum* YC1.2; 1, *L. plantarum* YC41; 2, *L. plantarum* APC2; 3, *L. plantarum* PG1; 4, *L. plantarum* PG2; 5, *L. plantarum* MNN1; 6, *L. plantarum* MC2; 7, *L. plantarum* YD1; 8, *L. plantarum* YD2.

One hundred three colonies exhibited clear zones on de Man-Rogosa-Sharpe (MRS) agar supplemented with 2% (wt/vol) CaCO_3_, and 21 strains were identified as *L. plantarum* by 16S rRNA gene sequencing (see Table S3 in the supplemental material). Among these 21 *L. plantarum* strains, 13 strains could not form any strand, while *L. plantarum* YC41, APC2, MNN1, MC2, PG1, PG2, YD1, and YD2 showed a ropy phenotype (Fig. 1A). The length of the strands was >1.2 cm. Notably, *L. plantarum* YC41 had the longest strand, which was >80 mm (Fig. 1A1). In addition, these strains with a ropy phenotype remained in suspension during incubation in MRS broth, whereas the nonropy strains were easier to aggregate, as evidenced by clarified medium with cells at the bottom (Fig. 1B). These results suggested that the eight *L. plantarum* strains could produce CPS.

### The *L. plantarum* strains with a ropy phenotype had a specific gene cluster for CPS biosynthesis.

**FIG 2:**
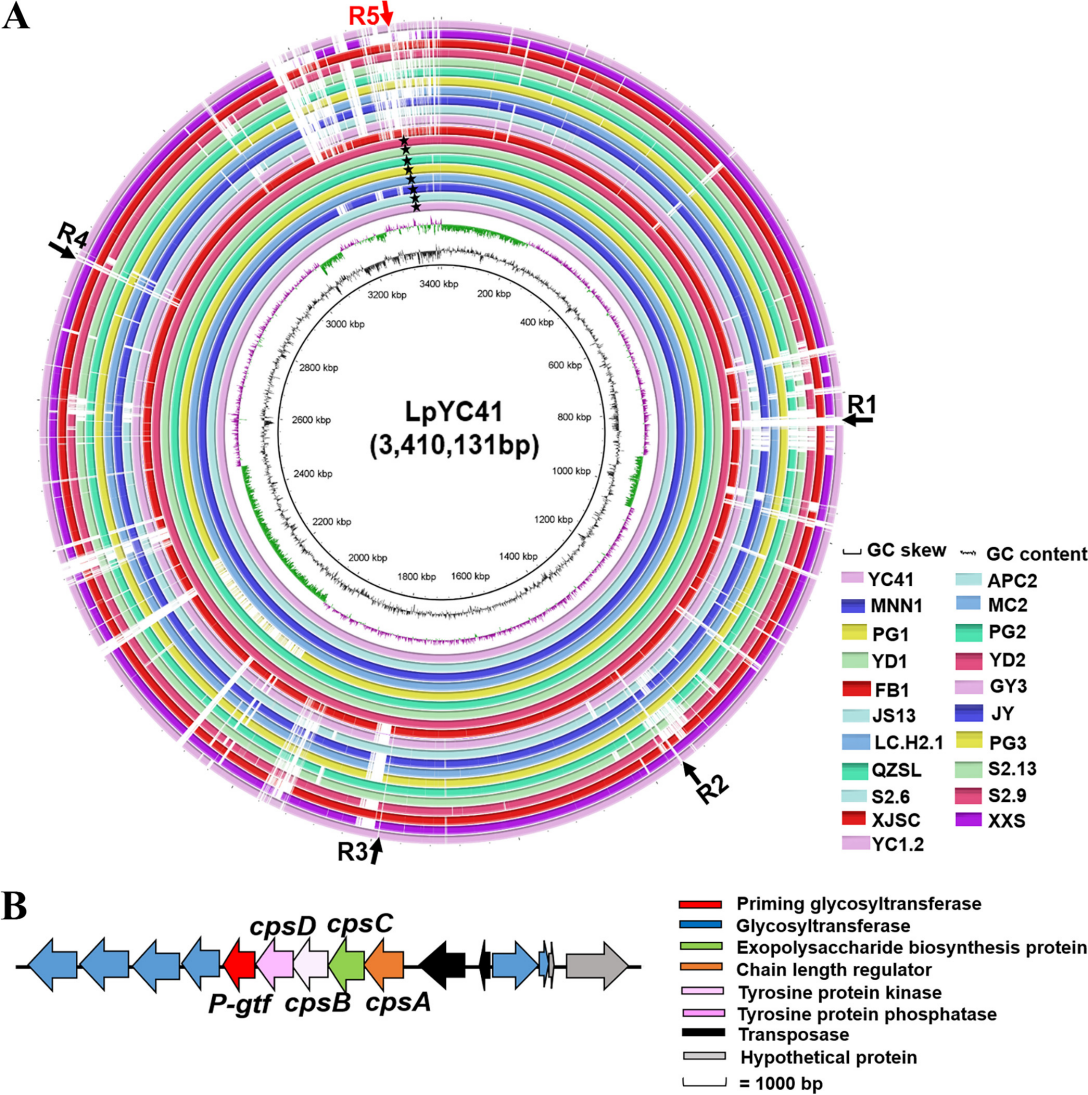
Comparative genomic analysis of 21 *L. plantarum* strains. (A) Comparative genomic analysis of 21 *L. plantarum* strains based on BLAST_VERSION+ bin analysis by BLAST Ring Image Generator v0.95. Matches with <50% identity or regions with no BLAST matches appear as blank spaces in each ring. The inner circle represents the reference sequence of *L. plantarum* YC41. Rings from the outermost to the center are nonropy strains YC1.2, XXS, XJSC, S2.9, S2.6, S2.13, QZSL, PG3, LC.H2.1, JY, JS13, GY3, and FB1 and ropy strains YD2, YD1, PG2, PG1, MC2, MNN1, APC2, and YC41, which are marked with black stars. R1 to R5 are five particular regions that were present only in the 8 ropy strains. R5 containing the putative CPS cluster is marked in red. (B) Physical map of the putative specific *cps* gene cluster in the 8 ropy strains.

To identify the genes responsible for the ropy phenotype, the draft genomes of 21 *L. plantarum* strains were sequenced in this study. The average nucleotide identities (ANIs) between YC41 and 7 ropy strains were >99.94%. However, the ANIs between YC41 and 13 nonropy strains were 98.98% to 99.20% (Table S5). It is worth noting that five particular regions, R1 to R5, were present only in the 8 ropy strains, which are the eight innermost circles in Fig. 2A and are marked with black stars. According to the gene annotation, R1, with 29,589 bp, contained 33 genes, where most of them encoded acyl carrier protein, 3-oxoacyl–acyl carrier protein reductase, 3-oxoacyl–acyl carrier protein synthase II, an acetyl-CoA carboxylase biotin carboxyl carrier protein subunit, acetyl-CoA carboxylase, biotin carboxylase, and acetyl-coenzyme A carboxylase carboxyl transferase subunit beta 2, etc. R2, with 10,858 bp, harbored 10 genes, which mainly encoded RNA degradosome polyphosphate kinase, K01514 exopolyphosphatase, and RNA polymerase (RNAP)-binding regulatory protein, etc. R3, with 6,573 bp, contained 9 genes, where most of them encoded hypothetical proteins. R4, with 12,571 bp, contained 9 genes, which mainly encoded hypothetical proteins. Notably, only R5, with 11,060 bp, included a series of genes involved in CPS biosynthesis. There were 14 genes in R5, including the *cpsA* gene encoding a LytR-CpsA-Psr (LCP) family protein, the *cpsB* gene encoding a tyrosine-protein phosphatase, the *cpsC* gene encoding a chain length regulator, the *cpsD* gene encoding a tyrosine-protein kinase, the *cpsE* gene encoding a priming glycosyltransferase, and 6 genes encoding glycosyltransferases (Fig. 2B). However, genes encoding flippase and polymerase were not found in this gene cluster, suggesting that this cluster might be incomplete. Therefore, complete genomic sequencing of *L. plantarum* YC41 was carried out in this study.

### The specific *cps* gene cluster *cpsYC41* is located in a novel plasmid, pYC41.

**FIG 3:**
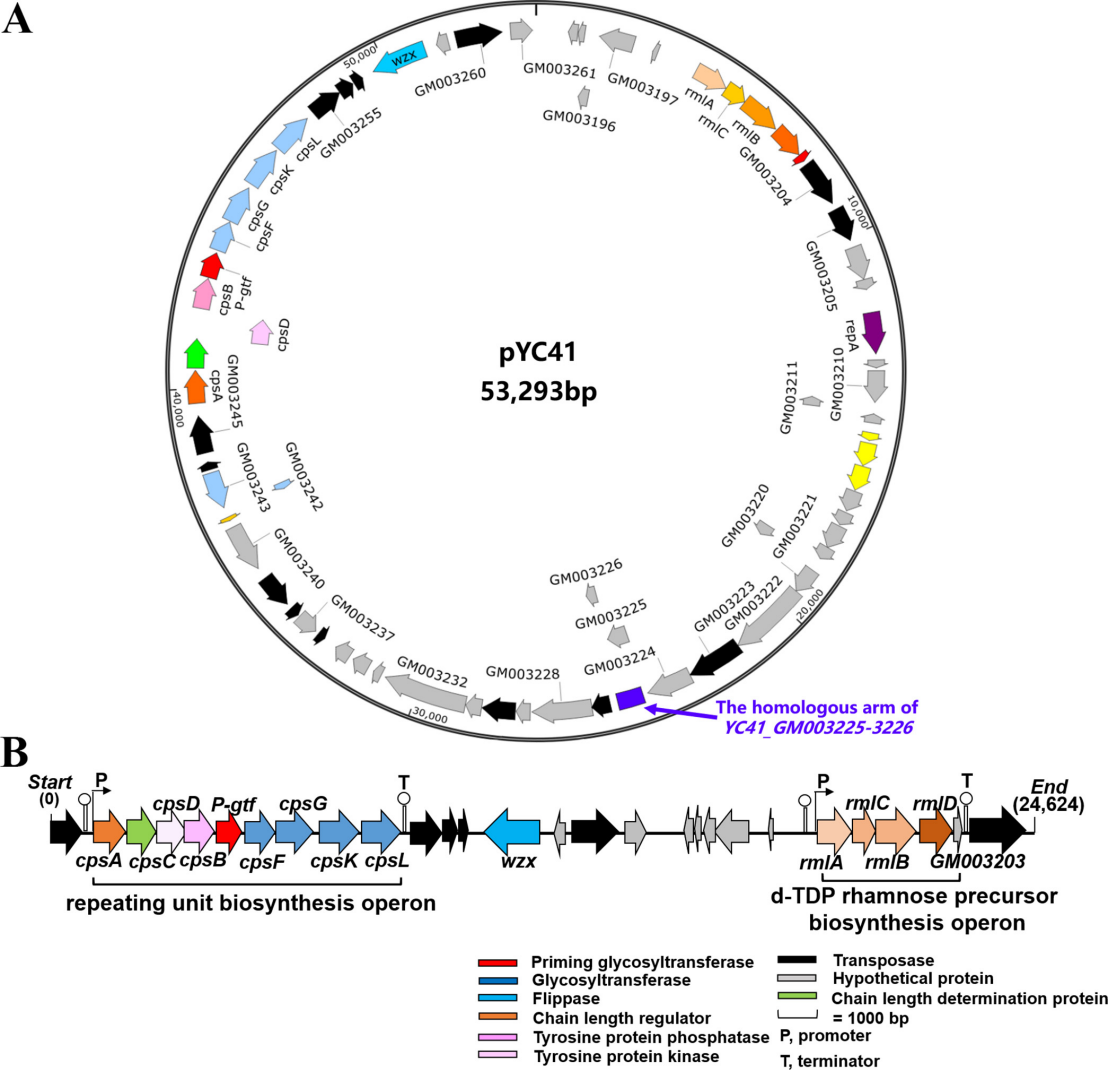
Gene organizations of the plasmid pYC41-borne CPS biosynthetic gene cluster. (A) Physical map and gene organization of plasmid pYC41. (B) Physical map of the *cpsYC41* gene cluster.

Complete genomic sequencing indicated that YC41 contained a 3,249,498-bp circular chromosomal DNA, with a G+C content of 44.65%. Notably, the *cpsYC41* gene cluster was located in a 53,923-bp plasmid with a G+C content of 39.14%, which was designated pYC41 (Fig. 3A). The nucleotide sequence of pYC41 showed <57% identity with other plasmids in GenBank, suggesting that it might be a novel plasmid. Sequence analysis showed that plasmid pYC41 had 68 open reading frames (ORFs), including the *repA* gene encoding replication initiator protein A, 26 genes involved in CPS biosynthesis, 14 genes encoding transposases, 3 genes encoding MobA/MobL family proteins, and genes encoding hypothetical proteins (Fig. 3A). In addition, a real-time quantitative PCR (qPCR) assay showed that the copy number of plasmid pYC41 was 2 copies in each cell (Fig. S4). Compared with single-copy genes in the chromosome, the expression levels of plasmid-borne genes can be increased due to the higher copy number (22, 23).

**FIG 4:**
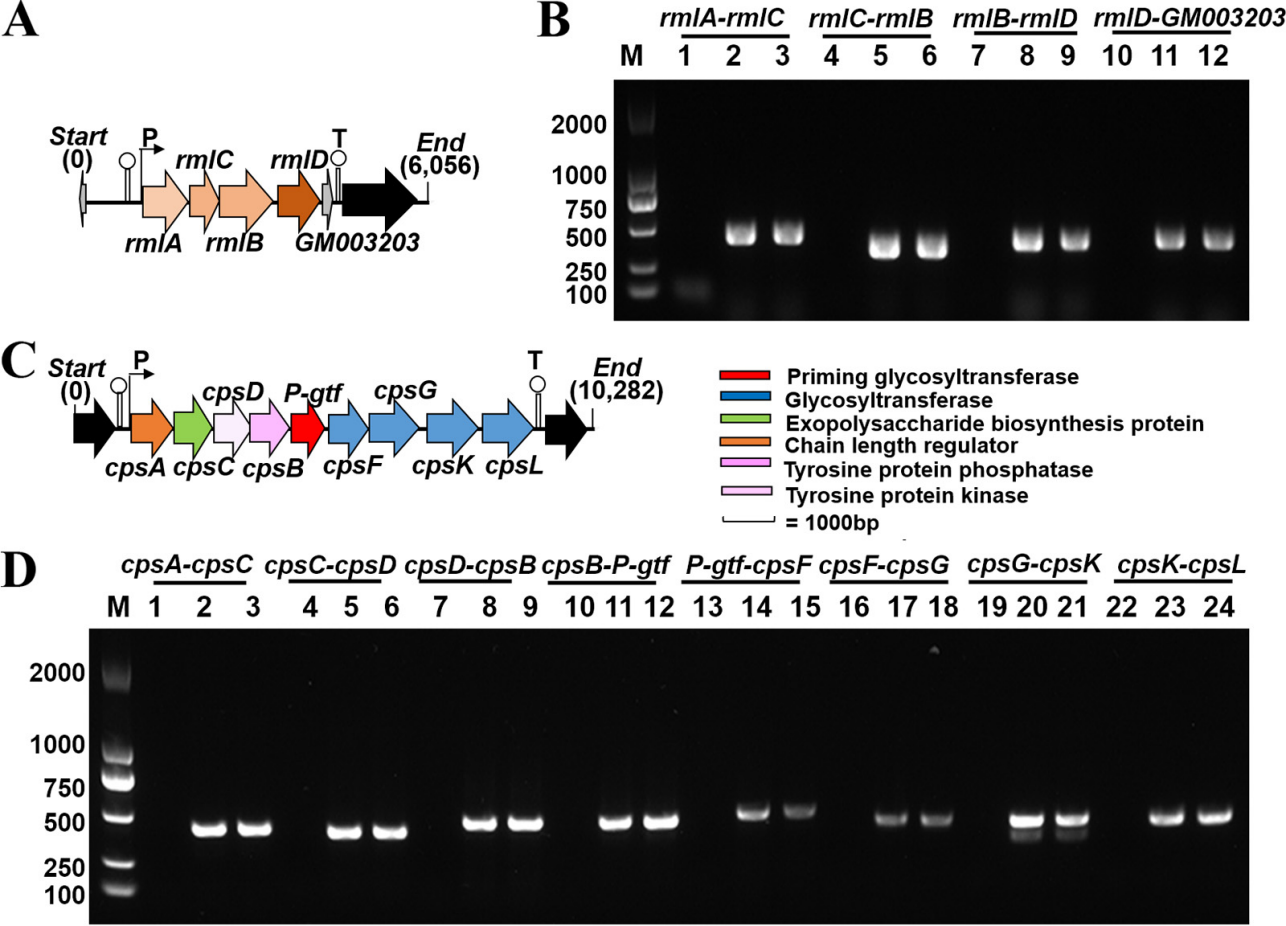
*In silico* analysis and RT-PCR assays to verify the cotranscription of the specific *cpsYC41* gene cluster. (A) Linear map of the dTDP-rhamnose precursor biosynthesis genes. (B) RT-PCR assays to verify the cotranscription of *rmlA* to *GM_3203*. Lane M, DNA marker (2,000 bp); lanes 1, 4, 7, and 10, negative controls omitting the reverse transcriptase in the RT reaction; lanes 2, 5, 8, and 11, positive controls of PCR using genomic DNA as the template; lanes 3, 6, 9, and 12, test groups with the reverse transcriptase in the RT reaction. (C) Linear map of the repeating-unit biosynthesis genes. (D) RT-PCR assays to verify the cotranscription of *cpsA* to *cpsL*. Lane M, DNA marker (2,000 bp); lanes 1, 4, 7, 10, 13, 16, 19, and 22, negative controls omitting the reverse transcriptase in the RT reaction; lanes 2, 5, 8, 11, 14, 17, 20, and 23, positive controls of PCR using genomic DNA as the template; lanes 3, 6, 9, 12, 15, 18, 21, and 24, test groups with the reverse transcriptase in the RT reaction.

The *cpsYC41* gene cluster was 22,074 bp in length and contained 26 putative ORFs (Fig. 3B). Based on amino acid sequence and protein structural similarity, putative functions were assigned to 26 genes within this gene cluster. Among them, the *cpsA* gene encoding an LCP domain-containing protein was assigned to regulation. The *cpsB* gene encoding a CpsD/CapB family tyrosine-protein kinase, the *cpsC* gene encoding a tyrosine-protein kinase transmembrane modulator, and the *cpsD* gene encoding a tyrosine-protein phosphatase were assigned chain-length control functions in CPS biosynthesis. The priming glycosyltransferase encoded by the *P-gtf* gene had a domain architecture similar to that of CpsE from Streptococcus agalactiae, which could transfer the first nucleotide sugar to a lipid carrier to initiate repeating-unit synthesis. The central region of the *cpsYC41* gene cluster encoded 4 glycosyltransferases, CpsF, CpsG, CpsK, and CpsL; the flippase Wzx; transposases; and several hypothetical proteins. In addition, the *rmlA* gene encoding glucose-1-phosphate thymidylyltransferase, the *rmlB* gene encoding dTDP-4-dehydrorhamnose 3,5-epimerase, the *rmlC* gene encoding dTDP-glucose 4,6-dehydratase, and the *rmlD* gene encoding dTDP-4-dehydrorhamnose reductase were assigned to dTDP-rhamnose precursor biosynthesis. However, no gene encoding the polymerase Wzy was found in the *cpsYC41* gene cluster. In fact, there was a gene encoding the polymerase Wzy in the *cps2* gene cluster in the chromosome of *L. plantarum* YC41 (Fig. S3). The *wzy* gene encoded a protein with high identity (97.41%) to the polysaccharide polymerase of the *cps4* gene cluster located in the chromosome of *L. plantarum* WCFS1. There may have been collaboration and communication of *cps* gene clusters in *L. plantarum* YC41 that contributed to capsular polysaccharide production. Notably, promoter and terminator predictions suggested that there were two operons in the *cpsYC41* gene cluster. A reverse transcription-polymerase chain reaction (RT-PCR) assay with cDNA as the template further suggested that genes *cpsA* to *cpsL* formed a polycistronic operon (Fig. 4C and D). Moreover, *rmlA*, *rmlC*, *rmlB*, *rmlD*, and *GM_3203* were cotranscribed and formed another operon in this gene cluster (Fig. 4A and B).

### The *cpsYC41* gene cluster was responsible for CPS biosynthesis.

In order to confirm the function of the *cpsYC41* gene cluster in CPS biosynthesis, a small internal portion of the *rmlA* or *cpsC* gene was cloned into a suicide plasmid, pUC19E. Next, homologous recombination led to insertional mutations and a polar effect on the operons. The resulting mutants were verified by PCR using the specific primers listed in Table S2. Sequencing showed that the PCR product consisted of the expected fragments and the erythromycin (Em) resistance gene, indicating the correct integration of the recombinant plasmid into the chromosome via single-crossover homologous recombination. In addition, the *YC41_GM003225*-*3226* genes were also inactivated to obtain the corresponding mutant as a control. The inactivation of *YC41_GM003225-3226* had no significant effects on cell growth and CPS yields (Fig. S6).

**FIG 5:**
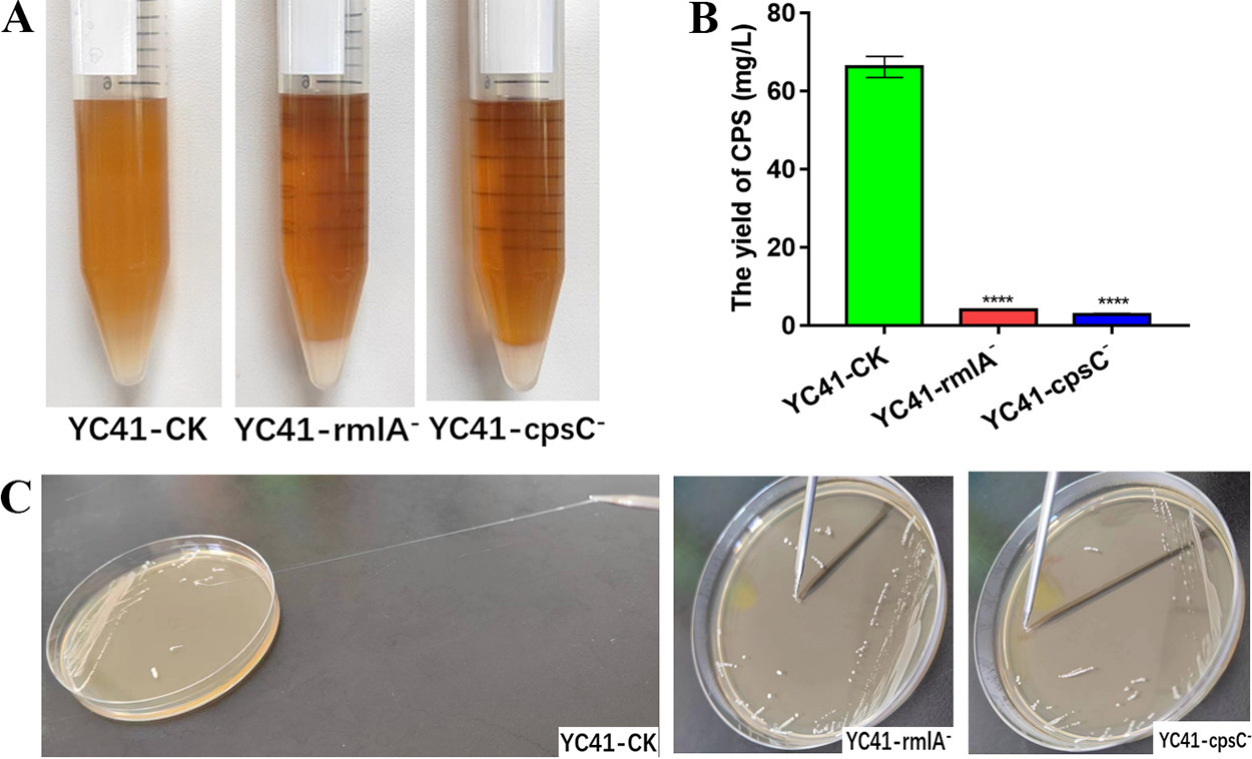
Functional analysis of the *cpsYC41* gene cluster. (A) Phenotypes of YC41-CK, YC41-rmlA^−^, and YC41-cpsC^−^ cultured for 20 h in MRS broth. (B) Yields of CPS in YC41-CK, YC41-rmlA^−^, and YC41-cpsC^−^. All results were obtained from at least three independent experiments. Data are reported as the means ± SD from three independent experiments (****, *P < *0.001). (C) Detection of the ropy phenotype in YC41-CK, YC41-rmlA^−^, and YC41-cpsC^−^ on MRS agar containing 2% (wt/vol) sucrose.

**FIG 6:**
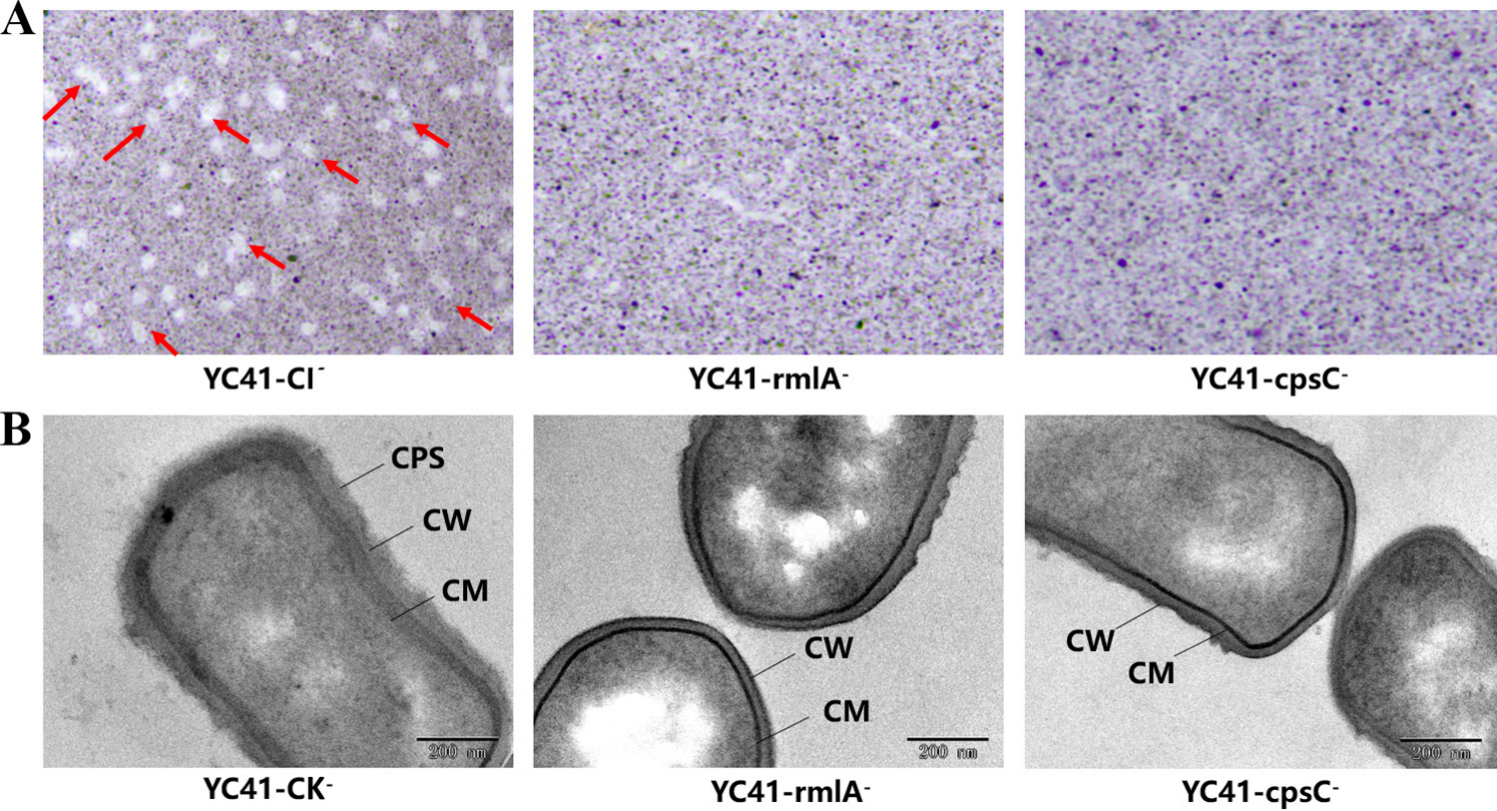
Functional analysis of the *cpsYC41* gene cluster. (A) Light microscopy observations of CPS in YC41-CK, YC41-rmlA^−^, and YC41-cpsC^−^ with India ink (magnification, ×1,000). (B) TEM images of YC41-CK, YC41-rmlA^−^, and YC41-cpsC^−^. CM, cell membrane; CW, cell wall.

During incubation in MRS broth, the YC41-rmlA^−^ and YC41-cpsC^−^ mutants could aggregate at the bottom of the tube, whereas the control strain YC41-control check (YC41-CK) remained in suspension (Fig. 5A). In addition, the colonies of YC41-rmlA^−^ and YC41-cpsC^−^ were not able to produce any strands when extended with an inoculation loop, while the colony of the control mutant had long strands (Fig. 5C). When fresh cultures grown overnight were mixed with India ink, clear halos could be observed around the cells of YC41-CK. However, no clear halos could be observed around the *rmlA*- and *cpsC*-deleted mutants, suggesting that the *cpsYC41* gene cluster was involved in CPS biosynthesis (Fig. 6A). Moreover, transmission electron microscopy (TEM) images showed that the control mutant had a visible outer layer formed by polysaccharides, whereas this layer was absent in the *rmlA*- and *cpsC*-deleted mutants (Fig. 6B). Notably, YC41-CK produced 66.19 mg/L polysaccharides, while the strains with inactivated *rmlA* and *cpsC* significantly reduced the polysaccharide yields to 4.11 and 2.90 mg/L, respectively (Fig. 5B). These results indicated that the *cpsYC41* gene cluster played a critical role in CPS biosynthesis and the ropy phenotype.

### The *cpsYC41* gene cluster was associated with CPS biosynthesis in other *L. plantarum* strains.

**FIG 7:**
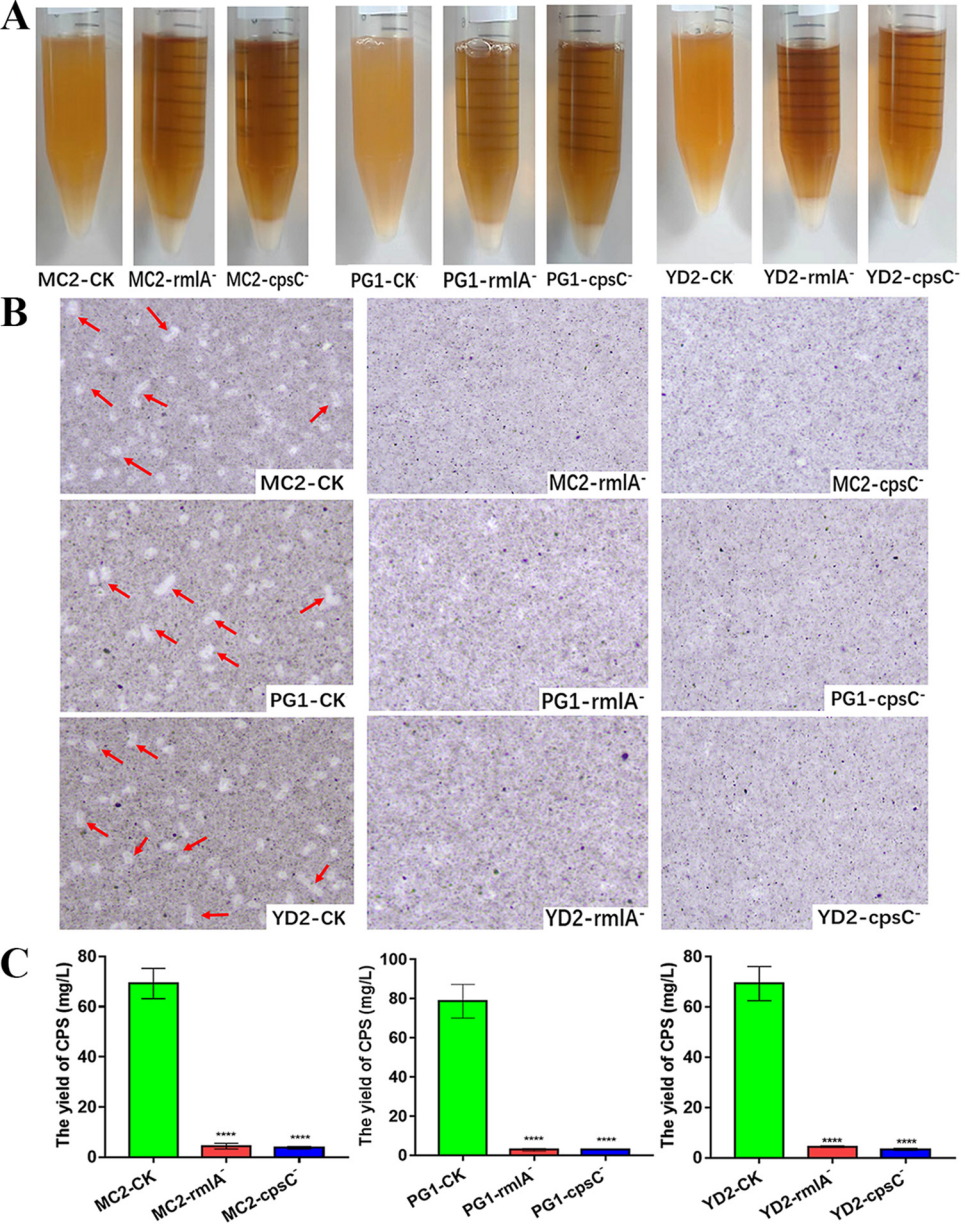
Further functional analysis of the *cpsYC41* gene cluster. (A) Phenotypes of YC41-CK, YC41-rmlA^−^, and YC41-cpsC^−^ cultured for 20 h in MRS broth. (B) Light microscopy observations of CPS in YC41-CK, YC41-rmlA^−^, and YC41-cpsC^−^ stained with India ink (magnification, ×1,000). (C) Yields of CPS in YC41-CK, YC41-rmlA^−^, and YC41-cpsC^−^. All results were obtained from at least three independent experiments. Data are reported as the means ± SD from three independent experiments (****, *P < *0.001).

In order to confirm the relationship between the *cps* gene cluster and CPS biosynthesis, the corresponding mutants were also obtained in other ropy *L. plantarum* strains such as MC2, PG1, and YD2. Compared with the wild-type strains, the cells of the corresponding *rmlA-* and *cpsC*-deleted mutants could aggregate after the stationary phase, and also, a clear halo could not be observed by capsule staining (Fig. 7A and B). In addition, the levels of CPS in *rmlA*- and *cpsC*-deleted mutants of other *L. plantarum* strains were also decreased by over 90% (*P < *0.001) (Fig. 7C). These findings indicated that the *cpsYC41* gene cluster was responsible for CPS biosynthesis in other ropy *L. plantarum* strains.

### CPS improved the resistance of YC41 to environmental stresses.

**FIG 8:**
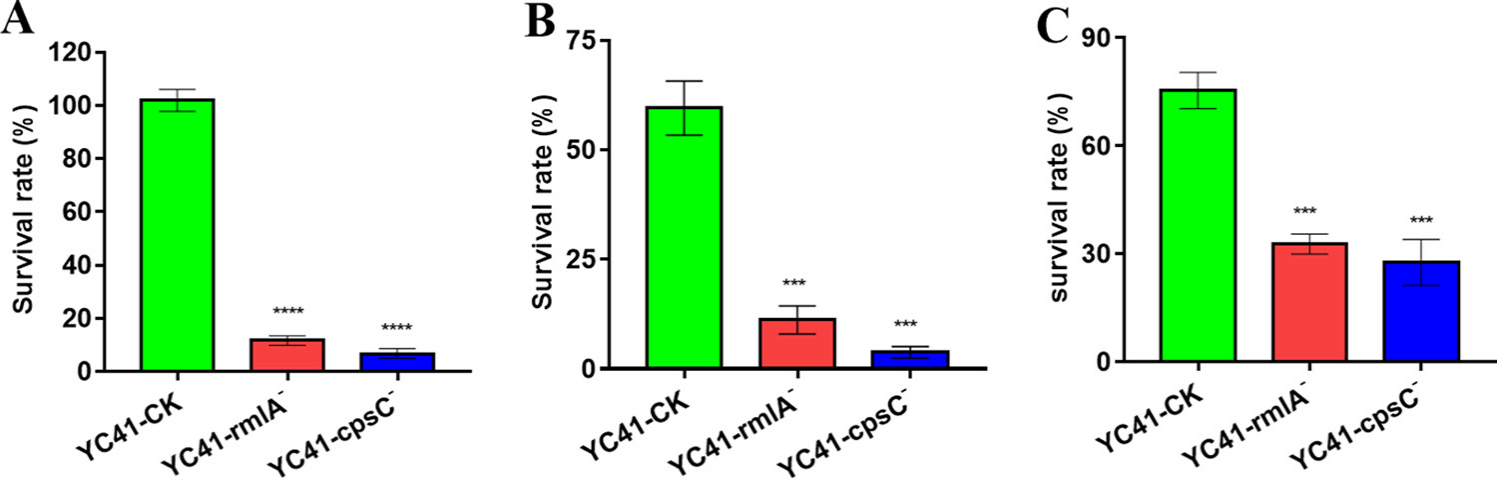
Resistance provided by the *cpsYC41* gene cluster. (A) Survival rates of YC41-CK, YC41-rmlA^−^, and YC41-cpsC^−^ in MRS broth at pH 3.0. (B) Survival rates of YC41-CK, YC41-rmlA^−^, and YC41-cpsC^−^ in MRS broth with 5% NaCl. (C) Survival rates of YC41-CK, YC41-rmlA^−^, and YC41-cpsC^−^ in MRS broth with 1.5 mM H_2_O_2_. All results were obtained from at least three independent experiments. Data are reported as the means ± SD from three independent experiments (***, *P < *0.001; ****, *P < *0.001).

In order to investigate whether CPS could protect YC41 against environmental stresses, the survival capacity was measured by subjecting stationary-phase cells to low pH, NaCl, and H_2_O_2_. The results showed that the survival rate of YC41-CK was maintained at 101.90% at pH 3.0. However, the survival rates of YC41-rmlA^−^ and YC41-cpsC^−^ were only 11.65% and 6.79%, respectively (*P < *0.001) (Fig. 8A). The survival rate of the control mutant reached 59.57% in the presence of 5% (wt/vol) NaCl, while the survival rates of the *rmlA*- and *cpsC*-deleted mutants were only 11.21 and 3.77%, respectively (*P < *0.001) (Fig. 8B). Under 1.5 mM H_2_O_2_ stress, the survival rate of YC41-CK was 75.40%, which was ~2-fold higher than those of the *rmlA*- and *cpsC*-deleted mutants (*P < *0.001) (Fig. 8C). These results showed that CPS could confer to *L. plantarum* YC41 enhanced tolerance to multiple environmental stresses.

## DISCUSSION

The complete genome sequencing of more than 180 *L. plantarum* strains enabled a better understanding of the gene clusters related to EPS biosynthesis. Four major EPS biosynthetic gene clusters have been identified in *L. plantarum*, which are designated *cps1*, *cps2*, *cps3*, and *cps4*. In the well-characterized *L. plantarum* strain WCFS1, four *cps* gene clusters are all present in the chromosomal DNA (18). In addition, some EPS biosynthesis gene clusters were located on mobile genetic elements such as plasmids in *L. plantarum* C410L1, HFC8, LZ227, ZJ102, and LTC-113 (16, 20, 21). Among these, only the molecular organization of the plasmid pYZ1-borne *cpsWc* gene cluster has been well characterized in *L. plantarum* LTC-113 (16). The *cpsWc* cluster was located in the 68.1-kb plasmid pYZ1 and contained 11 genes in an operon. Furthermore, this plasmid was confirmed to be responsible for CPS biosynthesis by plasmid-curing experiments (16). In this study, insertional inactivation experiments demonstrated that the *cpsYC41* gene cluster, which contained the dTDP-rhamnose precursor biosynthesis operon and the repeating-unit biosynthesis operon, was responsible for CPS biosynthesis. Sequence alignment by BLAST Ring Image Generator v0.95 (BRIG) showed that pYC41 had 53% similarity with plasmid unnamed3 (GenBank accession no. CP024061.1) of *L. plantarum* strain KACC 92189, whereas the coverage was only 53% (see Fig. S1 in the supplemental material). These results indicated that plasmid pYC41 was a novel plasmid harboring CPS biosynthetic gene clusters in *L. plantarum*.

In the *cpsYC41* gene cluster, the *rmlACBD* operon was highly conserved in some *L. plantarum* strains, which shared high nucleotide identity with *rfbACBD* in *L. plantarum* K25 plas3 (GenBank accession no. CP020096.1) (98.37%), *L. plantarum* 16 plasmid Lp16H (GenBank accession no. CP006041.1) (97.92%), and the *L. plantarum* G1 chromosome (GenBank accession no. CP053912.1) (98%), respectively (Fig. S2). Meanwhile, the *cpsACBD* operon showed 96.64% nucleotide identity with that in *L. plantarum* SKO-001 plasmid unnamed2 (GenBank accession no. CP040376.1) (Fig. S2). The gene encoding the flippase Wzx shared 99.44% nucleotide identity with the *wzx* gene in the chromosome of Limosilactobacillus fermentum HFD1 (GenBank accession no. CP050919.1) (Fig. S2). Moreover, the *wzx* gene was separated by two genes encoding transposases in the *cpsYC41* gene cluster. Taken together, there were also some mobile genetic elements, including 14 genes encoding transposases and 3 genes encoding MobA/MobL family proteins, flanking or within the *cpsYC41* gene cluster in pYC41. Therefore, the mosaic organization of the *cpsYC41* gene cluster probably resulted from horizontal gene transfer and homologous recombination events.

Capsular polysaccharide is known to enhance bacterial tolerance to harsh environmental stresses, maintaining the viability of the bacteria during industrial manufacturing (12, 24). Traditional Chinese sauerkraut is generally harvested in 2% to 10% salt brines at pH 3.0 (25, 26). CPS with the presence of OH groups can hold water around cells and further prevent dehydration to cope with osmotic stress (12, 27). When exposed to 5 mol/L NaCl, Lactobacillus mucosae DPC 6426, which could produce EPS, had a 2-fold increase in survival compared with the non-EPS-producing strain DPC 6420 (28). Under acid stress, CPS carrying negatively charged groups like phosphate groups can prevent proton diffusion into cells by the negative charge on the cell surface (12). A CPS-producing strain, *L. plantarum* LTC-113, had a 1.4-fold increase in tolerance to an acidic environment compared to its non-CPS-producing mutant (16). Our results also showed that the CPS-producing strain exhibited 15.0- and 15.80-fold increases in tolerance compared to the non-CPS-producing strain in the presence of low acid and 5% NaCl, respectively. In addition, *L. plantarum* strains suffer from oxidative stress during industrial processes. CPS can also scavenge harmful reactive oxygen species to prevent cell damage (29). When exposed to 20 mM H_2_O_2_, the cell counts of an EPS-deficient mutant declined by over 90% (30). Similarly, the survival rate of the CPS-producing strain was >2-fold higher than those of the *rmlA*- and *cpsC*-deleted mutants under 1.5 mM H_2_O_2_ stress in this study. Taken together, CPS is an important protection mechanism for bacteria to cope with and resist harsh environmental conditions. In addition, the increased gene copy number might also result in greater overall CPS production to protect the YC41 strain from environmental stresses (Fig. S4).

## MATERIALS AND METHODS

### Isolation and identification of *Lactobacillus* strains.

A total of 95 samples isolated mainly from fermented food, fruit, and meat were collected and serially diluted 10-fold in sterile 0.85% (wt/vol) NaCl. An aliquot of 0.1 mL of each dilution was plated onto de Man-Rogosa-Sharpe (MRS) agar with 2% CaCO_3_ and incubated at 37°C for 24 to 48 h. Colonies that produced a clear zone on MRS agar with CaCO_3_ were subjected to Gram staining. The 16S rRNA genes of Gram-positive *Lactobacillus* cultures were amplified by colony PCR using the primers listed in Table S2 in the supplemental material. The PCR products were sequenced by Sangon Biotech (Beijing, China) and analyzed by nucleotide BLAST analysis (http://blast.ncbi.nlm.nih.gov/Blast.cgi). The strains with high similarity to the 16S rRNA sequence of the *L. plantarum* reference strain (E value of 0 and maximum identity of ≥99%) were regarded as *L. plantarum* strains. The *L. plantarum* strains were maintained in MRS broth with 50% sterile glycerol and stored at −80°C (Table S3).

### Screening of CPS-producing *L. plantarum* strains.

Screening for the ability of *L. plantarum* isolates to produce CPS was performed according to methods described previously (31). Briefly, *L. plantarum* isolates were streaked onto MRS agar supplemented with 20 g/L sucrose and incubated at 37°C for 48 h. Colonies were dragged up using a sterile metal loop. If the length of the slime was more than 1.5 mm, the strains were considered CPS producers.

### Genome sequencing and comparative analysis.

The total cellular DNA was extracted using an E.Z.N.A. bacterial DNA kit according to the manufacturer’s instructions (Omega Bio-Tek Inc., Doraville, GA, USA). The draft genomes of 21 *L. plantarum* strains and the complete genome of YC41 were sequenced using the PacBio Sequel platform and the Illumina NovaSeq PE150 platform. Gene functions were predicted by GO (Gene Ontology), KEGG (Kyoto Encyclopedia of Genes and Genomes), and COG (Clusters of Orthologous Groups). Genome similarity analysis was carried out using OrthoANIu (available at https://www.ezbiocloud.net/tools/ani) (32). Comparative genomic analysis was conducted based on BLAST_VERSION+ bin analysis by BLAST Ring Image Generator v0.95 (BRIG) (33). Matches with <50% identity or regions with no BLAST matches appear as blank spaces in each ring. Accession numbers of the genomes deposited used in the present study are provided in Table S3. Functional annotation of the cluster region was carried out by combining the results of BLASTN, BLASTP, and CD-search of the Conserved Domain Database (CDD). Glycosyltransferases were predicted by the Carbohydrate-Active Enzymes database.

### Validation of the dTDP-rhamnose precursor biosynthesis operon and repeating-unit biosynthesis operon by reverse transcription-PCR.

Total RNA was extracted using an RNA extraction kit (Tiangen, Beijing, China) according to the manufacturer’s instructions and digested with RNase-free DNase I to remove DNA. RNA concentrations were quantified using a NanoDrop 2000 spectrophotometer (Thermo Fisher Scientific, Wilmington, DE, USA). Subsequently, reverse transcription was carried out using a HiScript III RT SuperMix for qPCR kit (Vazyme Biotech, Nanjing, China), with 1 μg of the total RNA as the template. Standard PCR was carried out using Q5 high-fidelity DNA polymerase (New England BioLabs, Ipswich, MA), with cDNA as the template. To determine whether adjacent genes were cotranscribed, specific primers amplifying the intergenic region between genes were used, as listed in Table S2. PCR products were analyzed by electrophoresis on a 1% (wt/vol) agarose gel and verified by DNA sequencing.

### Insertional inactivation of genes involved in CPS biosynthesis.

To study the role of the *cps* gene cluster in CPS biosynthesis, a series of mutants of *L. plantarum* YC41 was constructed using single-crossover homologous recombination, as shown in Fig. S5. Internal fragments of the target genes were amplified by PCR with the primers listed in Table S2 and inserted into the XbaI and KpnI sites of suicide plasmid pUC19EM (34). The recombinant plasmid was transformed into *L. plantarum* strains by electroporation. The insertional mutants were selected on MRS agar plates containing 10 μg/mL erythromycin (Em). To confirm integration at the correct locus, PCR was performed using the specific primers listed in Table S2, and the resulting amplicon was confirmed by sequencing. Meanwhile, the insertional inactivation of the *YC41_GM003225-3226* genes encoding hypothetical proteins was carried out using the same procedure to construct a control strain. Bacterial strains and plasmids are listed in Table S1.

### Growth assay and capsule staining of *L. plantarum* mutants.

Cultures of *L. plantarum* mutants and control strains grown overnight were inoculated (1%, vol/vol) into 50 mL MRS broth. Growth curves were determined by measuring the optical density at 600 nm or performing viable cell counts at 2-h intervals. For capsule staining, 10 μL of the fresh cultures grown overnight was mixed on a microscope slide with 10 μL of India ink and covered with a coverslip. The slide was examined using a Leica DM1000 microscope with an oil immersion lens at a ×1,000 magnification.

### Transmission electron microscopy assay.

Cultures grown overnight were fixed in 2.5% glutaraldehyde at 4°C for 14 h as described previously (35). After fixation, the cells were rinsed in 0.1 M phosphate buffer (pH 7.4). The pellets were then postfixed in 2% osmium tetroxide at 4°C for 2 h, dehydrated in a graded alcohol series followed by acetone, and embedded in resin. Then, the resin was transferred into Leica EM UC7 to prepare sections. Sections were stained with uranyl acetate followed by lead citrate for 5 min each and examined using the JEM1200EX transmission electron microscope (JEOL, Japan) at 100 kV.

### Isolation and quantification of CPS.

The extraction of CPS was carried out using a method reported previously (36), with minor modifications. Briefly, cultures grown overnight were centrifuged (8,000 × *g* for 20 min), washed twice, and then resuspended in sterile 1 mol/L NaCl. CPS was dissociated from the cells by sonication at 60 W for 5 min at 4°C. The proteins in the supernatant were precipitated by using 4% (vol/vol) trichloroacetic acid (TCA). The obtained supernatant was collected and precipitated with 75% ethanol at 4°C for 12 h, followed by centrifugation under the same conditions as the ones described above. The crude CPS was dialyzed with a membrane with a molecular weight cutoff of 14 kDa. The yield of CPS was estimated by the phenol-sulfuric acid method using glucose as a carbohydrate standard and expressed as milligrams per liter (37).

### Survival assay of *L. plantarum* mutants under acid, oxidative, and osmotic stresses.

Five milliliters of cultures grown overnight was collected and centrifuged at 6,000 × *g* for 3 min. Cell pellets were collected and washed twice with sterile phosphate-buffered saline (PBS) buffer. For acid stress assays, cell pellets were resuspended in 5 mL MRS broth adjusted to pH 3.0 and incubated at 37°C for 3 h. For osmotic stress assays, cell pellets were resuspended in 5 mL MRS broth supplemented with 5% (wt/vol) NaCl and incubated at 37°C for 24 h. For oxidative stress assays, cell pellets were resuspended in 5 mL MRS broth with 1.5 mM H_2_O_2_ and incubated at 37°C for 30 min. Survival for each treatment was determined by 10-fold serial dilutions and enumeration on MRS plates with erythromycin. The survival rate was calculated by dividing the viable cell counts after treatment by the value obtained before treatment. Three independent biological replicates were performed in this study.

### Determination of the relative plasmid copy number (PCN) by real-time PCR.

Amplification and detection were carried out in an ABI Step One Plus real-time PCR system (Applied Biosystems Inc.) using SYBR green qPCR master mix +genomic DNA (+gDNA) wiper (Vazyme Biotech, Nanjing, China). The alanine racemase gene (*alr*), a single-copy gene in the chromosome of *L. plantarum* WCFS1, was used as the reference gene (GenBank accession no. LP_RS02190 (Gene ID: 1061815)). A 219-bp fragment of the *alr* gene and a 147-bp fragment of the *repA* gene were separately amplified using the primers indicated in Table S2. The relative copy number of plasmid pYC41 was calculated as *N*_relative_ = (1 + *E*)^−Δ^*^CT^* (38), where *E* and Δ*C_T_* represent the PCR amplification efficiency and the difference between the threshold cycle number (*C_T_*) of the *alr* reaction and that of the *repA* reaction, respectively.

### Statistical analysis.

Data were analyzed using GraphPad Prism 6 software for Windows (GraphPad Software Inc., La Jolla, CA, USA). All data are reported as means ± standard deviations (SD). When two groups were compared, unpaired Student’s *t* test with Welch’s correction was used to calculate *P* values.

### Data availability.

The complete genome of YC41 was deposited in GenBank under accession no. CP101640. The accession numbers of the 21 newly assembled draft genomes were deposited at GenBank under the following accession numbers: JANEZN000000000, JANEYV000000000, JANEYX000000000, JANEZL000000000, JANEZI000000000, JANEZK000000000, JANEZG000000000, JANEYY000000000, JANEZH000000000, JANEZJ000000000, JANEYZ000000000, JANEZC000000000, JANEZD000000000, JANEZE000000000, JANEZF000000000, JANEZB000000000, JANEZM000000000, JANEZA000000000, JANFNB000000000, JANFNC000000000, JANEYW000000000. The detail genomic features of the 21 draft genomes are listed in Table S3.
