# Supplementary material for: Molecular Organization and Functional Analysis of a Novel Plasmid-Borne cps Gene Cluster from Lactiplantibacillus plantarum YC41
Source: Microbiol Spectr. 2023 Mar 6;11(2):e04150-22. doi: 10.1128/spectrum.04150-22 (PMC10100969; doi:10.1128/spectrum.04150-22)
Supplement: Supplemental file 1 — Supplemental material. Download spectrum.04150-22-s0001.pdf, PDF file, 0.8 MB [file spectrum.04150-22-s0001.pdf]

Supplementary Materials

Molecular organization and functional analysis of a novel plasmid-encoded *cps* gene cluster from *Lactiplantibacillus plantarum* YC41

Jieran An[1], Yuchen Zhang[1], Zhaoer Zhao[1], Ran Huan[1], Huaxi Yi[3], Hui Wang[1], Chunguang Luan[4], Shengbao Feng[5], Heqiang Huang[5], Shanwen Li[5], Deliang Wang [4], Zhengyuan Zhai[1*], Yanling Hao[2*]

[1] Key Laboratory of Functional Dairy, Co-constructed by Ministry of Education and Beijing Municipality, College of Food Science and Nutritional Engineering, China Agricultural University, Beijing, China

[2] Key Laboratory of Precision Nutrition and Food Quality, Department of Nutrition and Health, China Agricultural University, Beijing, China

[3] College of Food Science and Engineering, Ocean University of China, Qingdao, China

[4] China National Research Institute of Food and Fermentation Industries, Beijing, China

[5] Qinghai Huzhu Barley Wine Co. Ltd, Haining, China

***Corresponding authors:**

Zhengyuan Zhai, zhaizy@cau.edu.cn Yanling Hao, haoyl@cau.edu.cn

TABLE S1 Bacteria strains and plasmids used in this study.

Strains and plasmids	Relevant characteristics	Source or reference
**Strains**		
YC41	Host strain	Sauerkraut, Henan
MC2	Host strain	Sausage, Xinjiang
PG1	Host strain	Apple, Beijing
YD2	Host strain	Vagina, Beijing
E.coli DH5α	F^{-}ϕ80d*lac*ZΔM15, Δ (*lacZYA–argF*) U169, *deoR, recA1, endA1, hsdR17* (r_K^-, m_K^-), *phoA, supE44, λ⁻, thi-1, gyrA96, relA1*. Host strain for pUC vectors.	TIANGEN
YC41-CK⁻	*L. plantarum* YC41 with *YC41_GM003225-3226* interrupted	This work
YC41-rmlA⁻	*L. plantarum* YC41 with *rmlA* interrupted	This work
YC41-cpsC⁻	*L. plantarum* YC41 with *cpsC* interrupted	This work
MC2-CK⁻	*L. plantarum* MC2 with *MC2_GM003140-3141* interrupted	This work
MC2-rmlA⁻	*L. plantarum* MC2 with*rmlA* interrupted	This work
MC2-cpsC⁻	*L. plantarum* MC2 with *cpsC* interrupted	This work
PG1-CK⁻	*L. plantarum* PG2 with *PG2_GM003234-3235* interrupted	This work
PG1-rmlA⁻	*L. plantarum* PG2 with *rmlA* interrupted	This work
PG1-cpsC⁻	*L. plantarum* PG2 with *cpsC* interrupted	This work
YD2-CK⁻	*L. plantarum* YD2 with *YD2_GM003229-3230* interrupted	This work
YD2-rmlA⁻	*L. plantarum* YD2 with *rmlA* interrupted	This work
YD2-cpsC⁻	*L. plantarum* YD2 with *cpsC* interrupted	This work
**Plasmids**		
pUC19EM	Suicide plasmid carried a Em^R cassette, derivative of pUC19 Amp^R, Em^R	Ma *et al*., 2019
pUCEM-*CK*	pUC19EM containing partial sequence of *control* genes	This work
pUCEM-*rmlA*	pUC19EM containing partial sequence of *rmlA*	This work

pUCEM-*cpsC*	pUC19EM containing partial sequence of *cpsC*	This work

Amp^{R}, ampicillin resistance; *Em*^{R}, erythromycin resistance.

TABLE S2 Primers used in this study.

**Primers**	**Sequence (5'-3')**	**Restriction enzymes**	**Purposes**
16S- 27F	AGAGTTTGATCCTGGCTCAG		16S rDNA
16S-1492R	TACGGTTACCTTGTTACGACTT		
CK-F	CGCTCTAGATCGCTTACAACAGCAAAAGC	*Xba*I	Amplify the 666bp fragment of control genes
CK-R	CGGGGTACCAATCCGGGCAATTCATCGC	*Kpn*I	
rmlA-F	CGCTCTAGACACCTGTTGATACACCACG	*Xba*I	Amplify the 580bp fragment of *rmlA*
rmlA-R	CGGGGTACCTTTAAGTTCTGGCGTTTTTTG	*Kpn*I	
cpsC-F	CGCTCTAGAGCAACTACTGAAATTCTG	*Xba*I	Amplify the 439bp fragment of *cpsC*
cpsC-R	CGGGGTACCGCTCCAGCCAGAGTAAATAG	*Kpn*I	
EM-F	TTCCTGAGCCGATTTCAAAGAT		Confirm the integration of pUC *CK/rmlA/cpsC*
CK-gR	GGTACTGACGTTAATTGATATTTTTGAATG		
rmlA-gR	GCCCTCCCAATTAATCTTAATAGTATTGAC		
cpsC-gR	GGTTGTGTCCATCAATTCCTT		
repA-F	GTGCCGAACTCCACAACA		Determine the relative copy number of pYC41
repA-R	CGTCTCAAGGCTTTAGTCATC		
alr-F	TATGAACGCCGATTACAAGG		
alr-R	CTAACCCACAAGCAATCTCAT		

TABLE S3 Genomic features and Accession numbers of newly assembled draft genomes of 21 *L. pantarum* strains.

Strain	Source	Genome size (bp)	G-C (%)	Gene number	tRNA	rRNA	sRNA	Accession no.
YC41	Sauerkraut, Henan	3,410,131	44.27	3,307	66	9	5	JANEZN000000000
APC2	Pickle, Beijing	3,407,521	44.28	3,305	66	9	5	JANEYV000000000
GY3	Pickle, Sichuan	3,304,246	44.45	3,198	65	9	5	JANEYX000000000
XXS	Sauerkraut, Shanxi	3,140,836	44.68	3,054	61	9	1	JANEZL000000000
S2.9	Pickle, Sichuan	3,205,851	44.34	3,152	65	9	4	JANEZI000000000
XJSC	Sauerkraut, Xinjiang	3,321,747	44.31	3,201	65	9	5	JANEZK000000000
QZSL	Silage, Beijing	3,348,735	44.37	3,254	68	9	0	JANEZG000000000
JS13	Sauerkraut, Gansu	3,310,436	44.39	3,187	67	9	3	JANEYY000000000
S2.6	Koumiss, Xinjiang	2,942,659	44.83	2,844	61	9	5	JANEZH000000000
S2.13	Milk, Beijing	3,147,635	44.51	3,070	68	9	4	JANEZJ000000000
JY	Milk, Tianjin	2,944,507	44.83	2,845	61	9	5	JANEYZ000000000
MNN1	Yak milk	3,550,577	44.33	3,448	66	9	4	JANEZC000000000
PG1	Apple, Beijing	3,415,343	44.26	3,313	66	9	5	JANEZD000000000
PG2	Apple, Shandong	3,403,018	44.29	3,300	66	9	4	JANEZE000000000
PG3	Apple, Hebei	3,223,080	44.46	3,116	69	9	0	JANEZF000000000
MC2	Sausage, Xinjiang	3,331,962	44.42	3,230	66	9	5	JANEZB000000000
YC1.2	Meat, Hainan	3,535,208	44.15	3,481	71	8	4	JANEZM000000000

LC.H2.1	Sausage, Liaoning	3,299,379	44.4	3,217	73	9	6	JANEZA000000000
YD1	Vagina, Beijing	3,409,700	44.28	3,308	66	9	5	JANFNB000000000
YD2	Vagina, Beijing	3,412,410	44.28	3,310	68	9	7	JANFNC000000000
FB1	Human feces, Beijing	3,398,887	44.22	3,302	66	9	4	JANEYW000000000

Table S4 Protein homology analysis of gene products of the *L. plantarum* YC41 *cps* gene clusters.

**Cluster and ORF**	**Gene**	**Protein size (aa)**	**Predicted encoded function**	**Best blastp match**		**Identity (%)**
				**Protein (accession no.)**	**Organism**	
***cps1* gene cluster**						
YC41_GM001086	*cps1F*	302	glycosyltransferase family 2 protein	glycosyltransferase (WP_053338797)	*Lactiplantibacillus*	99.67
YC41_GM001087	*cps1G*	310	glycosyltransferase family 2 protein	glycosyltransferase family 2 protein (WP_021357577)	*Lactiplantibacillus plantarum*	100
YC41_GM001088	*glf2*	393	UDP-galactopyranose mutase	UDP-galactopyranose mutase (EFK29285.1)	*Lactiplantibacillus plantarum* ATCC 14917	99.75
YC41_GM001089	*cps1L*	385	hypothetical protein	polysaccharide biosynthesis protein (WP_224272040)	*Lactiplantibacillus plantarum*	100
YC41_GM001090	*cps1M*	207	hypothetical protein	polysaccharide biosynthesis protein (WP_223843879.1)	*Lactiplantibacillus plantarum*	100
YC41_GM001091	*cps1N*	391	hypothetical protein	polysaccharide biosynthesis protein (WP_196241911.1)	*Lactiplantibacillus plantarum*	99.74

YC41_GM001092	*cps1O*	369	polysaccharide biosynthesis protein	polysaccharide biosynthesis protein (WP_076640318.1)	*Lactiplantibacillus plantarum*	100
YC41_GM001093	*Cps1I*	359	acyltransferase family protein	acyltransferase family protein (WP_003643284.1)	*Lactiplantibacillus plantarum*	100
YC41_GM001094	*Cps1K*	258	DUF4422 domain-containing protein	DUF4422 domain-containing protein (WP_021356757)	*Lactiplantibacillus plantarum*	99.61
YC41_GM001095	*Wzx*	472	polysaccharide biosynthesis C-terminal domain-containing protein	polysaccharide biosynthesis C-terminal domain-containing protein (WP_076640316.1)	*Lactiplantibacillus plantarum*	100
YC41_GM001096	*P-gtf*	222	priming glycosyltransferase	Priming glycosyltransferase (ACT61928.1)	*Lactiplantibacillus plantarum* JDM1	100
***cps2* gene cluster**						
YC41_GM001900		92	polysaccharide biosynthesis protein, chain length regulator	Wzz/FepE/Etk N-terminal domain-containing protein （WP_256633518.1）	*Clostridioides difficile*	100
YC41_GM001899		159	polysaccharide biosynthesis protein	hypothetical protein (WP_255781095.1)	*Lactiplantibacillus plantarum*	100
YC41_GM001898	*csp2D*	226	CpsD/CapB family tyrosine-protein kinase	CpsD/CapB family tyrosine-protein kinase (WP_072540686.1)	*Lactiplantibacillus plantarum*	100
YC41_GM001897	*csp2B*	264	tyrosine protein phosphatase	Polysaccharide biosynthesis protein (AGE39648.1)	*Lactiplantibacillus plantarum* ZJ316	99.62

YC41_GM001896	*cps2I*	313	SDR family NAD(P)-dependent oxidoreductase	SDR family NAD(P)-dependent oxidoreductase (WP_003640785.1)	*Lactiplantibacillus*	100
YC41_GM001895	*P-gtf*	221	Undecaprenyl- phosphate glucose phosphotransferase	priming glycosyltransferase (ACT62648.1)	*Lactiplantibacillus plantarum* JDM1	99.55
YC41_GM001894	*cps2F*	362	glycosyltransferase family 4 protein	glycosyltransferase family 4 protein (WP_072540684.1)	*Lactiplantibacillus*	100
YC41_GM001893	*cps2G*	342	glycosyltransferase	glycosyltransferase (WP_033608772.1)	*Lactiplantibacillus plantarum*	100
YC41_GM001892	*wzy*	424	hypothetical protein	polysaccharide polymerase (WP_070085078.1)	*Lactiplantibacillus plantarum*	99.76
YC41_GM001891	*cps2K*	322	glycosyltransferase	glycosyltransferase (WP_021356070.1)	*Lactiplantibacillus plantarum*	100
YC41_GM001890	*cps2L*	47	hypothetical protein	Nd	Nd	Nd
YC41_GM001889	*wzx*	440	polysaccharide biosynthesis C-terminal domain-containing protein	polysaccharide biosynthesis C-terminal domain-containing protein (WP_033608769.1)	*Lactiplantibacillus plantarum*	100
***cpsYC41* gene cluster**						
YC41_GM003246	*cpsA*	273	LCP family protein	LytR family transcriptional regulator (MCG0715588.1)	*Lactiplantibacillus plantarum*	95.12
YC41_GM003247	*cpsC*	246	exopolysaccharide biosynthesis protein	exopolysaccharide biosynthesis protein (MCG0715589.1)	*Lactiplantibacillus plantarum*	99.56
YC41_GM003248	*cpsB*	250	CpsD/CapB family tyrosine-	CpsD/CapB family tyrosine-protein	*Lactiplantibacillus plantarum*	99.56

			protein kinase	kinase (MCG0715590.1)		
YC41_GM003249	*cpsD*	258	tyrosine protein phosphatase	tyrosine protein phosphatase (MCG0715591.1)	*Lactiplantibacillus plantarum*	98.84
YC41_GM003250	*P-gtf*	216	sugar transferase	sugar transferase (MCG0715592.1)	*Lactiplantibacillus plantarum*	99.07
YC41_GM003251	*cpsF*	257	glycosyltransferase	glycosyltransferase (WP_195546395.1)	*Lacticaseibacillus paracasei*	98.05
YC41_GM003252	*cpsG*	318	glycosyltransferase family 2 protein	glycosyltransferase family 2 protein (RDF96571.1)	*Lactiplantibacillus plantarum* KMB_618	99.06
YC41_GM003253	*cpsH*	351	EpsG family protein	EpsG family protein (MCG0715595.1)	*Lactiplantibacillus plantarum*	99.72
YC41_GM003254	*cpsI*	353	glycosyltransferase family 4 protein	glycosyltransferase family 4 protein (WP_114648661.1)	*Lactiplantibacillus plantarum*	99.72
YC41_GM003255		277	IS30 family transposase	IS30 family transposase (WP_208192194.1)	*Lactiplantibacillus plantarum*	100
YC41_GM003256		129	IS256 family transposase	transposase (WP_211742767.1)	*Lactiplantibacillus plantarum*	99.22
YC41_GM003257		82	transposase	transposase (WP_143448105.1)	*Levilactobacillus brevis*	100
YC41_GM003258	*wzx*	475	flippase	flippase (WP_137629754.1)	*Lactiplantibacillus daowaiensis*	99.79
YC41_GM003259		102	hypothetical protein	hypothetical protein (WP_181815124.1)	*Lactiplantibacillus plantarum*	100
YC41_GM003260		392	IS256 family transposase	IS256 family transposase (WP_063696917.1)	*Lactobacillaceae*	99.74
YC41_GM003261		184	recombinase family protein	recombinase family protein	*Lactobacillaceae*	100

				(WP_013356291.1)		
YC41_GM003194		80	hypothetical protein	hypothetical protein A8704_15460 (ANJ15465.1)	*Lactiplantibacillus plantarum*	100
YC41_GM003195		56	hypothetical protein	hypothetical protein (WP_181073324.1)	*Lactiplantibacillus plantarum*	98.21
YC41_GM003196		102	hypothetical protein	hypothetical protein (WP_003554871.1)	*Lactobacillaceae*	100
YC41_GM003197		295	hypothetical protein	AAA family ATPase (WP_076640261.1)	*Lactiplantibacillus plantarum*	100
YC41_GM003198		40	hypothetical protein LBP_p2g041	KRN39678.1	*Lactiplantibacillus plantarum*	100
YC41_GM003199	*rmlA*	289	glucose-1-phosphate thymidylyltransferase RfbA	glucose-1-phosphate thymidylyltransferase RfbA (WP_216491241.1)	*Lactiplantibacillus argentoratensis*	99.65
YC41_GM003200	*rmlC*	193	dTDP-4-dehydrorhamnose 3,5-epimerase	dTDP-4-dehydrorhamnose 3,5-epimerase (WP_114648650.1)	*Lactiplantibacillus plantarum*	100
YC41_GM003201	*rmlB*	342	dTDP-glucose 4,6-dehydratase	dTDP-glucose 4,6-dehydratase (WP_015825388.1)	*Lactiplantibacillus plantarum*	99.71
YC41_GM003202	*rmlD*	278	dTDP-4-dehydrorhamnose reductase	dTDP-4-dehydrorhamnose reductase (WP_057785044.1)	*Lactobacillaceae*	99.64
YC41_GM003203		64	sugar transferase	sugar transferase (WP_114648648.1)	*Lactiplantibacillus plantarum*	100

Nd, not detected.

Table S5 The ANI analysis of YC41 and other 20 *L. plantarum* strains.

	**Strain**	**Average Nucleotide Identity (ANI) (%)**
ropy strains	APC2	99.98
	MC2	99.94
	MNN1	99.94
	PG1	99.97
	PG2	99.98
	UD1	99.95
	YD2	99.95
non-ropy strains	FB1	99.14
	GY3	99.04
	JS13	98.98
	JY	98.98
	LC.H2.1	99.04
	PG3	99.14
	QZSL	99.00
	S2.6	98.96
	S2.9	98.82
	S2.13	99.04
	XJSC	99.13
	XXS	99.20
	YC1.2	99.04

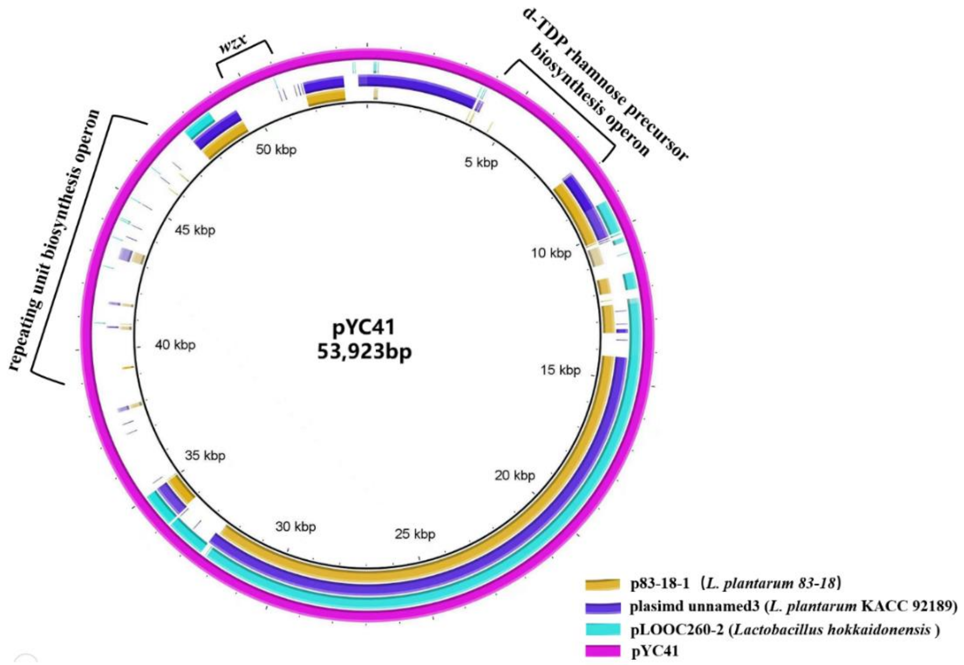

FIG S1 Comparative genomic analysis of the plasmid pYC41 with other plasmids.

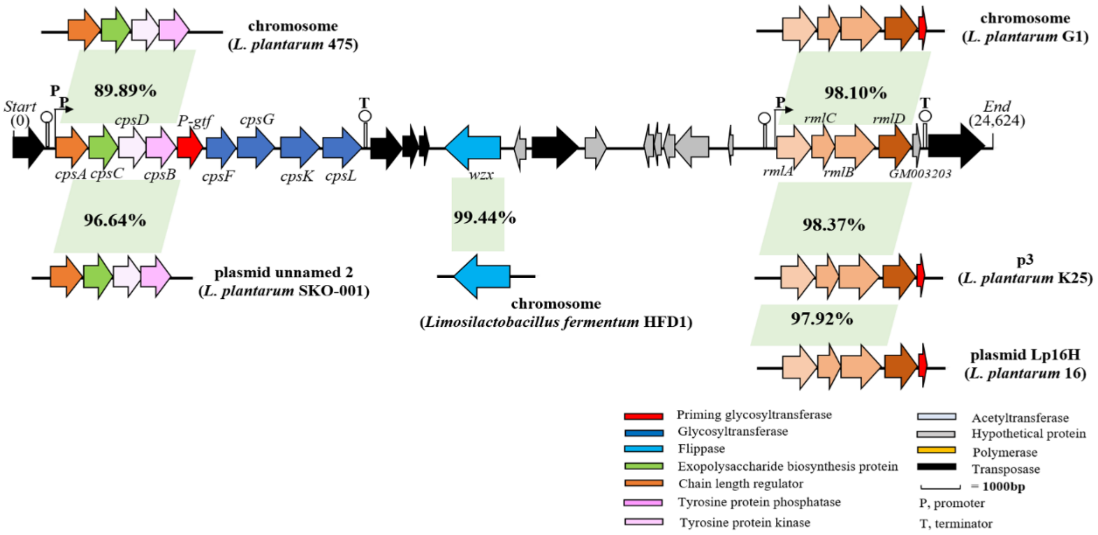

FIG S2 Multiple nucleotide homology analysis of the *cpsYC41* gene cluster.

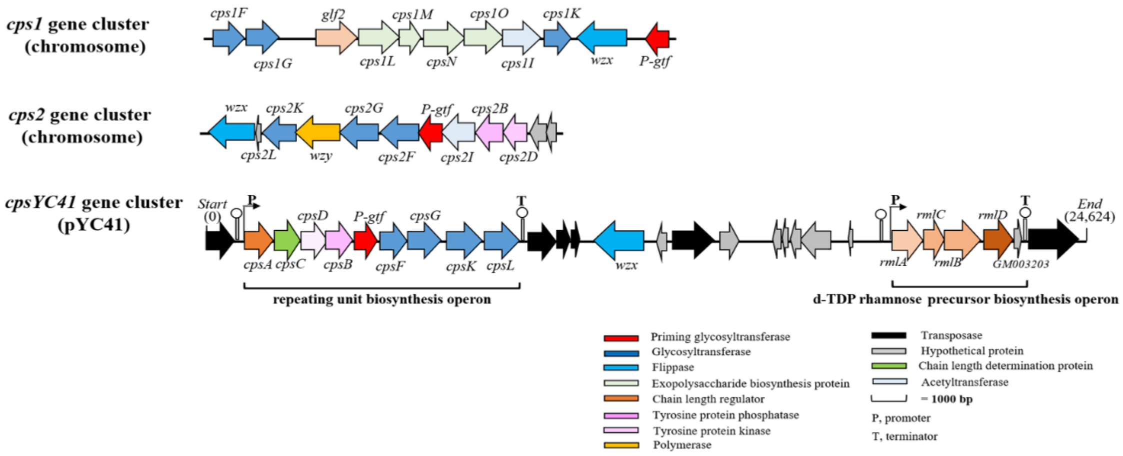

FIG S3 The physical map of *cps* gene clusters in *L. plantarum* YC41.

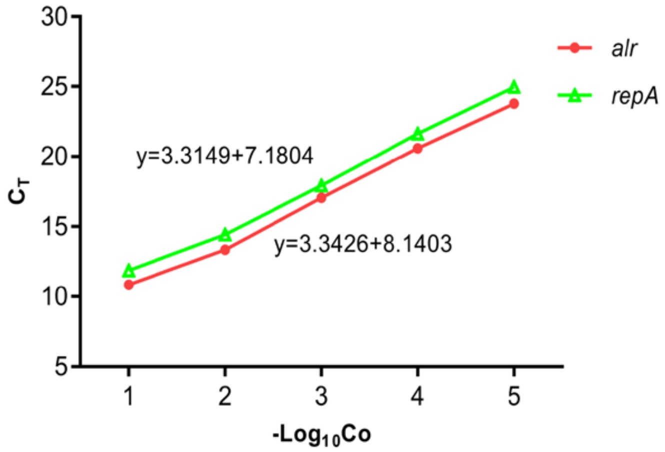

FIG S4 The standard curves of CT values versus template concentration for gene alanine racemase (*alr*, a single-copy gene) and gene *repA* (encoding replication initiator

protein A). The total DNA of *L. plantarum* YC41 was serial tenfold diluted, ranging from 10^{-1} to 10^{-5}, and the CT values of each gene were plotted against the logarithm of concentration (n = 3). A standard curve was generated by linear regression through these points for each gene.

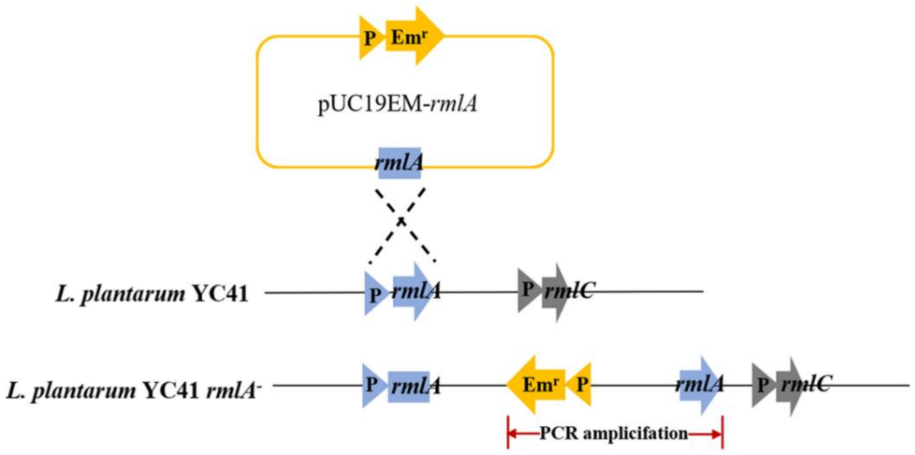

FIG S5 Construction of the *L. plantarum* mutants. Taking *rmlA* as an example, genes are represented by arrows, promoters are indicated by triangles, and the internal fragment of *rmlA* is represented by a solid bule box. Chromosomal DNA is represented by black lines, plasmid DNA is represented by yellow lines, and the red arrow indicates the PCR products amplified using the forward primer EM-F and the reverse primer rmlAg-R.

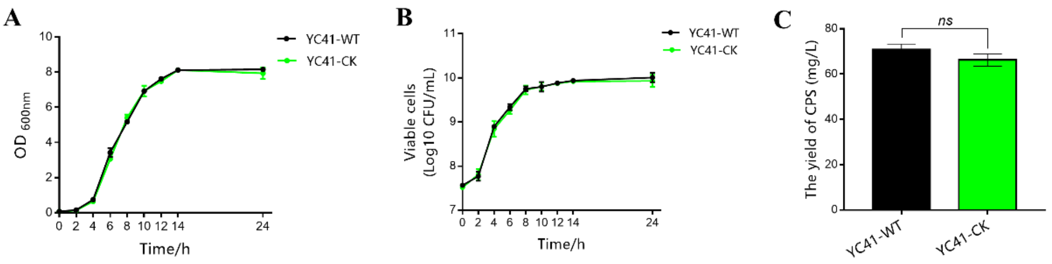

FIG S6 Biochemical characterizations of YC41-WT and YC41-CK. (A) The growth curve of YC41-WT and YC41-CK. (B) The viable cells of YC41-WT and YC41-CK. (C) The yield of CPS of YC41-WT and YC41-CK.
